# Intestinal inflammation promotes neuroinflammation and PD-associated nigrostriatal pathology independently of LRRK2 G2019S kinase activity

**DOI:** 10.3389/fncel.2026.1794784

**Published:** 2026-04-10

**Authors:** Andrea R. Merchak, Mary K. Herrick, Madelyn C. Houser, Cody E. Keating, Jianjun Chang, Malú Gámez Tansey

**Affiliations:** 1 Department of Neuroscience, University of Florida College of Medicine, Gainesville, FL, United States; 2 Department of Neurology, Stark Neuroscience Research Institute, Indiana University School of Medicine, Indianapolis, IN, United States; 3 Department of Physiology, Emory University School of Medicine, Atlanta, GA, United States; 4 Department of Cell Biology, Emory University School of Medicine, Atlanta, GA, United States

**Keywords:** circulating cytokines, dopaminergic (DA) neuron, G2019S BAC overexpression, intestinal inflammation, neuroinflammation

## Abstract

**Introduction:**

The pathogenesis of Parkinson’s disease (PD) has been linked to environmental factors, toxins, genetics, and peripheral inflammation. Importantly, intestinal inflammation like that seen in Crohn’s disease (CD) or food allergies has been implicated in risk for neurodegeneration and late-onset PD. Further, CD and PD share genetic risk factors including gain-of-function leucine-rich repeat kinase 2 (*LRRK2*) mutations. Here, we aim to better understand how intestinal inflammation synergizes with *Lrrk2* levels or kinase activation to promote neurodegeneration in young and old mice.

**Methods:**

We utilized bacterial artificial chromosome (BAC) mice overexpressing wildtype mouse *Lrrk2* or mutant G2019S mouse *Lrrk2* and compared them with C57B6J mice at baseline and under conditions of intestinal inflammation using dextran sodium sulfate (DSS) colitis models.

**Results:**

While our data revealed regulation of the brain inflammatory state by *Lrrk2*, we did not observe age-dependent selective vulnerability or protection in *Lrrk2* mouse lines in colitis protocols. Instead, DSS phenotypes were associated with increased nigrostriatal dysregulation in all genotypes independent of age.

**Discussion:**

While *Lrrk2* mutations appear to influence the genesis of peripheral inflammation, our data suggest that *Lrrk2* activation due to a gain-of-function mutation does not exacerbate the effects of inflammation on nigrostriatal degeneration in this model.

## 1 Introduction

Parkinson’s disease (PD) is a progressive movement disorder affecting more than one million Americans and over 10 million people worldwide, making it the second most common neurodegenerative disorder. PD is neuropathologically characterized by aggregation of α-synuclein (αsyn) leading to Lewy body formation and progressive loss of dopaminergic (DA) neurons in the substantia nigra pars compacta (SNpc) (Fahn, 2003). Loss of DA in the nigrostriatal pathway results in stereotypic motor symptoms associated with PD (von Campenhausen et al., 2005; Wirdefeldt et al., 2011). While the exact mechanisms have yet to be defined, several studies implicate the role of inflammation in the pathophysiology of PD. Late-onset PD has been associated with variations in the human leukocyte antigen (HLA) gene that encodes major histocompatibility complex (MHC) proteins necessary for antigen presentation (Hamza et al., 2010). Reduced CD4:CD8 T cell ratios are present in the blood of individuals with PD while T cell infiltration and activated microglia have been identified in the SNpc and in animal models (McGeer et al., 1988; Baba et al., 2005; Whitton, 2007; Saunders et al., 2012). Furthermore, increased brain inflammatory cytokines, (TNF, IFNγ, IL-1β) have been associated with increased oxidative stress and accelerated DA neurodegeneration in PD (Mogi et al., 1994; Blum-Degena et al., 1995; Brodacki et al., 2008; Reale et al., 2009). In addition to the stereotypical motor symptoms, PD patients also exhibit non-motor symptoms, with one of the most common of these being gastrointestinal (GI) dysfunction. An estimated 60–80% of PD patients experience GI symptoms (Siddiqui et al., 2002; Ueki and Otsuka, 2004; Chen et al., 2015). Moreover, studies suggest chronic constipation manifests in some PD patients at least 15 years before diagnosis, making it one of the earliest indicators of PD (Abbott et al., 2001; Savica et al., 2009; Postuma et al., 2013; Chen et al., 2015). In addition, PD patients exhibit increased intestinal permeability or “leaky gut,” with the level of permeability associated with increased intestinal pro-inflammatory cytokines (Forsyth et al., 2011; Devos et al., 2013). This may be in part due to infection or sustained exposure to microbial products resulting in intestinal inflammation (Mulak and Bonaz, 2015; Houser and Tansey, 2017). Therefore, we and others have proposed that intestinal inflammation and altered intestinal permeability contribute to systemic inflammation that in turn promotes neuroinflammation and neurodegeneration associated with PD pathogenesis (Mulak and Bonaz, 2015; Houser and Tansey, 2017).

Interestingly, PD shares characteristics with Crohn’s disease (CD), an inflammatory bowel disease (IBD) characterized by chronic relapsing inflammation of the GI tract. CD risk has been associated with over 160 genetic loci, several of which are related to inflammatory genes (*NOD2*, *TLR4*, *IL-23R*, *HLA*, and *TNF*) (Cario and Podolsky, 2000; Hugot et al., 2001; Ogura et al., 2001; Duerr et al., 2006; Burton et al., 2007; Barrett et al., 2008). Of these, 72 are shared with PD, most in domains affecting MHC (Kang et al., 2022). Similar to PD, CD patients exhibit increased levels of circulating pro-inflammatory cytokines (TNF, IFNγ, IL-12) (Murch et al., 1993; Daig et al., 1996; Fuss et al., 1996) and altered intestinal permeability that can contribute to intestinal inflammation (Gerova et al., 2011; Petit et al., 2012; Bischoff et al., 2014). Interestingly, patients with CD have a 28% increased risk of PD which declines with anti-inflammatory drug treatment (Zhu et al., 2019). In 2008, studies identified leucine-rich repeat kinase 2 (*LRRK2*) as a susceptibility locus for CD (Barrett et al., 2008; Franke et al., 2010). *LRRK2* is one of the greatest genetic contributors to PD, with the *LRRK2* G2019S mutation being the most prevalent. This and other mutations that increase risk for PD reside in the kinase domain and result in a 2–3-fold increase in kinase activity (West et al., 2005, 2007; Greggio et al., 2006). A recent genome-wide association study (GWAS) identified new *LRRK2* variants, N2081D and N551K/R1398H as risk and protective genetic variants, respectively, for both CD and PD (Hui et al., 2018). The N2081D variant resides in the same kinase domain as the G2019S mutation and results in an increase in kinase activity albeit not to the same extent as the G2019S mutation (Hui et al., 2018). The N2081D mutation leads to increased RAB10 phosphorylation while the G2019S preferentially leads to phosphorylation of RAB12. The result of this difference is that N2018D is more closely associated with inflammatory disorders and colitis than the G2019S mutation which is more closely associated with PD (Heaton et al., 2025). These reports highlight the mutation-specific changes that cannot simply be described by increased LRRK2 kinase activity.

While pathogenic mechanisms underlying the association between PD and *LRRK2* mutations are not well understood, LRRK2 has been shown to regulate inflammatory processes (Wallings and Tansey, 2019; Herrick and Tansey, 2021; Wallings et al., 2023, 2024), and our group has shown its protein expression is increased in peripheral blood immune cells from PD patients relative to age- and sex-matched controls (Cook et al., 2017). Interestingly, another group has shown that *LRRK2* levels are increased in immune cells in inflamed tissue from CD patients (Gardet et al., 2010). It’s been proposed that increased LRRK2 kinase activity due to increased wildtype (WT) protein expression or the G2019S mutation drives dysregulation resulting in increased inflammatory cytokines (Moehle et al., 2012, 2015; Lee et al., 2017). A handful of studies have examined the role of LRRK2 in experimental models of intestinal inflammation (Yan et al., 2022; Cabezudo et al., 2023; Wang et al., 2024). One report suggests bacterial artificial chromosome (BAC) transgenic mice overexpressing wildtype *Lrrk2* are more susceptible to dextran sodium sulfate (DSS)-induced colitis than wildtype mice, due in part to an increase in kinase activity which leads to dysregulated inflammatory signaling (Takagawa et al., 2018). Another study found that the G2019S *Lrrk2* mutation resulted in higher susceptibility to colitis and neurodegeneration (Lin et al., 2022). However, studies of the effects of WT or mutant *Lrrk2* in colitis models have been limited in scope and do not compare the effects of overexpression of wildtype *Lrrk2* compared to the mutant *Lrrk2* found in patients. Thus, we aim to better understand how the G2019S mutation modulates the inflammatory response to DSS and whether it synergizes with the inflammation to induce neurodegeneration. We use a BAC LRRK2 overexpression mouse model to examine the effects of increased LRRK2 protein and increased G2019S-mediated kinase activity on the GI system and the central nervous system (CNS) after intestinal inflammation (Houser and Tansey, 2017). We hypothesized that G2019S-mediated kinase activity synergizes with intestinal inflammation to promote GI dysfunction and PD-like neuroinflammation and neuropathology in the CNS. Understanding the effects of LRRK2 and its kinase activity on the CNS and GI system under conditions of peripheral inflammation will shed critical insight in development of immunomodulatory neuroprotective therapies to prevent, delay or slow progression of PD pathologies while avoiding collateral damage on the immune system.

## 2 Materials and methods

### 2.1 Animals

Homozygous male BAC *Lrrk2-G2019S* (B6.Cg-Tg(Lrrk2*G2019S)2Yue/J; stock number 012467) and BAC *Lrrk2-WT* (B6.Cg-Tg(Lrrk2)6Yue/J; stock number 012466) BAC transgenic mice were purchased from the Jackson Laboratory and bred to hemizygosity at Emory University. Hemizygous male and female BAC transgenic mouse strains overexpressing either mouse mutant G2019S *Lrrk2* (G2019S) or mouse wildtype *Lrrk2* (WTOE) were used for experimental procedures with non-transgenic littermates on the same genetic background (B6/J) serving as controls. Genotypes were determined by tail-snip PCR with two sets of primers: Transgene: Forward 5′ GAC TAC AAA GAC GAT GAC GAC AAG 3′ Reverse 5′ CTA CCA CCA CCC AGA TAA TGT C 3′; Internal positive control: Forward 5′ CAA ATG TTG CTT GTC TGG TG 3′ Reverse 5′ GTC AGT CGA GTG CAC AGT TT 3′. Animals were group-housed (maximum 5 mice per cage) and maintained on a 12 h/12 h light/dark cycle with *ad libitum* access to standard rodent chow and water. Experimental procedures involving use of animals were performed in accordance with the National Institutes of Health Guidelines for Animal Care and Use and approved by the Institutional Animal Care and Use Committee at Emory University School of Medicine.

### 2.2 Experimental timeline

Male and female B6, BAC WTOE, and G2019S mice were subjected to different DSS-induction paradigms. For acute DSS-induced colitis and recovery, 2–3-month-old male and female B6, WTOE, and G2019S mice were subjected to 5 days of 2% DSS in the drinking water followed by 5 days of autoclaved tap water *ad libitum* (*n* = 18–26 per genotype). For the aged paradigm, 16–18-month-old B6 and G2019S mice were subjected to the same acute DSS-induced colitis paradigm (*n* = 12–19 per genotype). For chronic DSS-induced colitis, 2–3-month-old male and female B6, WTOE, and G2019S mice were subjected to 1.5% DSS in the drinking water for 5 days followed by autoclaved tap water for 5 days. This was repeated three consecutive times for a total of 30 days (*n* = 19–22 per genotype). Water controls were placed on autoclaved tap water for their respective study durations. Mice were weighed and assessed for disease activity indexes (DAI) daily for acute paradigms or every other day for the chronic paradigm.

### 2.3 Tissue collection

At the end of the paradigm, mice were euthanized by decapitation. Brain tissue was removed, with the right hemisphere designated for flow cytometry. From the left hemisphere, the striatum, SN, and cortex were dissected, flash frozen and stored in −80 °C until processing. For the acute paradigm, caudal tissue from the left hemisphere containing the SN was post-fixed in 4% paraformaldehyde for 24 h at 4 °C for immunohistochemistry.

### 2.4 Plasma and peripheral blood mononuclear cell isolation

Immediately prior to sacrifice, whole blood was collected by submandibular bleeds with approximately 200 μL per mouse collected. Whole blood was collected in ethylenediaminetetraacetic acid (EDTA) coated tubes (Covidien 8881311248). Fifty microliters of whole blood was treated with 1× red blood cell (RBC) lysis buffer (BioLegend 420301) to lyse RBCs as per the manufacturer’s instructions prior to staining PBMCs for flow cytometry. The remaining 150 μL of whole blood was centrifuged, after which plasma was removed and promptly frozen on dry ice then subsequently stored at −80 °C until processing.

### 2.5 Brain immune cell isolation

Brain immune cell isolation was performed as previously described with minor modifications (MacPherson et al., 2017). The right hemisphere was minced in 1× HBSS (without calcium, magnesium, and phenol red, Invitrogen, 14175) and transferred to an enzymatic digestion solution consisting of 1.4 U/mL collagenase, type VIII (Sigma, C2139), 1 mg/mL DNase 1 (Sigma, DN25) in RPMI1640 medium (ThermoFisher, 11875085). The tissue and enzymatic solution were incubated at 37 °C for 15 min with shaking every 5 min. The solution was then neutralized with 10% FBS (heat inactivated, Atlanta Biologicals, S11150) in RPMI1640 medium and centrifuged to pellet tissue. The remaining tissue pellet was homogenized with glass pipets in ice cold 1× HBSS and then filtered through a 70 μm cell strainer. After centrifugation, the remaining pellet was resuspended in 37% Percoll (Sigma, P1644). 70% Percoll was layered below the resuspended pellet while 30% Percoll was layered above. The Percoll gradient was centrifuged for 30 min at room temperature without a brake. Immune cells were isolated from the interface between the 37 and 70% layers, washed with 1× HBSS and then stained for flow cytometric analysis.

### 2.6 Flow cytometry

Fifty microliters of PBMCs were stained with BMV109 (1 μM, Vergent Bioscience, 40200-100) for 1 h at 37 °C. After a brief wash, PBMCs were then stained for 20 min with the following panel: live/dead fixable red dead cell stain (1:2,000, Invitrogen, 23102), anti-mouse CD16/CD32 (1:100, eBioscience, 14-0161-085), anti-mouse CD45 PerCP-Cy5.5 (1:100, eBioscience, 45-0451-80), anti-mouse CD19 BV650 (1:100, Biolegend, 115541), anti-mouse CD3 BV421 (1:50, Biolegend 100227), anti-mouse CD4 V500 (1:100, BD Biosciences, 560783), anti-mouse CD8 BV785 (1:100, Biolegend 100749), anti-mouse CD11b PE-Cy7 (1:200, Biolegend, 101215), anti-mouse MHCII APC-Cy7 (1:200, Biolegend, 107627), anti-mouse CD44 PE (1:200, Biolegend, 103007), anti-mouse Ly6G AF700 (1:100, eBioscience, 56-5931-80), anti-mouse CD11c BV711 (1:200, Biolegend, 117349), and anti-mouse Ly6C AF488 (1:100, Biolegend, 53-5932) in FACS buffer. Samples were fixed in 1% PFA for 30 min. Ten microliters of counting beads (AccuCheck Counting Beads, Invitrogen PCB100) were added to each sample. Samples were then run on an LSRII cytometer (BD Biosciences) and analyzed with FlowJo_V10 software.

Brain immune cells were stained with BMV109 (1 μM, Vergent Bioscience, 40200-100) and Pepstatin A, BODIPY FL Conjugate (1 μg/mL, Thermo Fisher Scientific, P12271) for 1 h at 37 °C. After a brief wash, brain immune cells were then stained for 20 min with the following panel: live/dead fixable red dead cell stain (1:2,000, Invitrogen, 23102), anti-mouse CD16/CD32 (1:100, eBioscience, 14-0161-085), anti-mouse CD45 PerCP-Cy5.5 (1:100, eBioscience, 45-0451-80), anti-mouse CD3 BV421 (1:50, Biolegend 100227), anti-mouse CD4 V500 (1:100, BD Biosciences, 560783), anti-mouse CD8 PE (1:100, eBioscience, 12-0083-81), not anti-mouse CD11b PE-Cy7 (1:200, Biolegend, 101215), anti-mouse MHCII APC-Cy7 (1:200, Biolegend, 107627), anti-mouse Ly6C BV785 (1:100, Biolegend 128041), anti-mouse CD86 BV605 (1:50, Biolegend, 105037), anti-mouse Ly6G AF700 (1:100, eBioscience, 56-5931-80), and anti-mouse CD11c BV711 (1:200, Biolegend, 117349) in FACS buffer. Samples were fixed in 1% PFA for 30 min. Ten microliters of counting beads (AccuCheck Counting Beads, Invitrogen PCB100) were added to each sample. Samples were then run on an LSRII cytometer (BD Biosciences) and analyzed with FlowJo_V10 software.

Example gating can be found in Supplementary Figure 1. For PBMCs, leukocytes were gated on side scatter area (SSC-A) (granularity) by forward scatter area (FSC-A) (size) and then with forward scatter height (FSC-H) by FSC-A to identify single leukocytes. To identify live CD45^+^ cells, the fixable red negative population was selected followed by the CD45^+^ population against FSC-H. CD45^+^ cells were then gated on CD19 by CD3, with CD3^+^ cells denoting T cells. T cells were then gated for CD4^+^ and CD8^+^ T cells to differentiate T helper cells vs. cytotoxic T cells, respectively. The CD19 B cell population was examined for MHCII expression by histogram and geometric mean fluorescent intensity. The CD19^−^, CD3^−^ population was gated on CD11b by CD19 to select CD11b^+^ positive cells. That population was then selected and gated for CD11b vs. Ly6G to identify neutrophils (CD11b^+^, Ly6G^+^). The non-neutrophil population was then gated for the Ly6C by MHCII cascade that distinguishes monocyte and macrophage populations (Ly6C^+^, MHCII- inflammatory monocytes, Ly6C^+^, MHCII^+^ monocytes, Ly6C^−^, MHCII^+^ non-inflammatory monocytes, and Ly6C^−^, MHCII^−^ monocytes). In each immune subset, the pan-cathepsin probe, BMV109, were examined for positive cells and geometric mean fluorescent intensity.

For brain immune cells, leukocytes were gated on SSC-A (granularity) by FSC-A (size) and then with FSC-H by FSC-A to identify single leukocytes. To identify live cells, the Fixable Red negative population was selected against FSC-H. From a CD45 by CD11b gate, lymphocytes and mixed monocytes/macrophages were distinguished from microglia populations. The microglia population was further analyzed for MHCII^+^ microglia and CD86^+^ microglia. The geometric mean fluorescent intensity of MHCII^+^ microglia or CD86^+^ microglia was also identified based on the respective antibody. The lymphocyte population was gated on CD3^+^ T cells followed by CD4^+^ and CD8^+^ T cells to differentiate T helper cells vs. cytotoxic T cells, respectively. The mixed monocyte/macrophage population was then examined with CD11b by Ly6G to distinguish neutrophils. The non-neutrophil gate was then gated with the Ly6C by MHCII cascade that distinguishes monocyte and macrophage populations (Ly6C^+^, MHCII^−^ inflammatory monocytes, Ly6C^+^, MHCII^+^ monocytes, Ly6C^−^, MHCII^+^ non-inflammatory monocytes, and Ly6C^−^, MHCII^−^ monocytes).

### 2.7 Multiplexed immunoassays

Levels of inflammatory protein were measured in plasma by the Emory Multiplexed Immunoassay Core (EMIC) using a commercially available V-plex Pro-inflammatory Panel 1 Mouse Kit per the manufacturer’s instructions on the Meso Scale Discovery QuickPlex (Meso-Scale Discovery, Gaithersburg, MD). Plasma samples were analyzed for cytokines and chemokines (IFNγ, IL-1β, IL-2, IL-4, IL-5, IL-6, IL-10, IL-12p70, KC/GRO, and TNF) that play key roles in the inflammation response and immune system. Samples were run in replicates of 30 μL by an experimenter blinded to treatment history and genotype.

### 2.8 RNA extraction and protein isolation

RNA extraction and protein isolation were performed as previously described (de Sousa Rodrigues et al., 2016). Tissue was homogenized in ice-cold TRIzol (Life Technologies, 15596018) using a stainless-steel bead (Qiagen, 69989) and a TissueLyser II (Qiagen, 85300). RNA was then isolated using QIAshredder columns (Qiagen, 79656) and RNeasy mini kits (Qiagen, 74106) according to the manufacturer’s protocol. RNA was quantified and purity assessed using a NanoDrop 2000 spectrophotometer (Thermo Fisher Scientific). Protein was isolated from the organic layer of the TRIzol separation method by methanol precipitation. Protein was resuspended in 1% SDS, quantified using a bicinchoninic acid (BCA) protein assay, and then used for immunoblotting.

### 2.9 cDNA synthesis and quantitative real-time polymerase chain reaction

Following the manufacturer’s protocol, RNA was reverse transcribed using SuperScript II Reverse Transcriptase (Life Technologies, 18064014), random hexamers (Integrated DNA Technologies, 51-01-18-25), and dNTPs (Life Technologies, 18427013). qPCR was performed as previously described (de Sousa Rodrigues et al., 2016). Using an ABI Prism 7900 HT Fast Real-time PCR System (Applied Biosystems), 25 ng cDNA was analyzed in triplicate with SYBR Green PCR Master Mix and 150 nM validated forward and reverse oligonucleotide primers (Integrated DNA Technologies). Cycle of threshold (Ct) values were normalized to the housekeeping gene *Gapdh.* Relative mRNA expression was obtained by subtracting normalized Ct values from a standard number. Primers used: *Gapdh*—Forward 5′ CAA GGT CAT CCA TGA CAA CTT TG 3′ Reverse 5′ GGC CAT CCA CAG TCT TCT GG 3′; *Snca*—Forward 5′ AAA TGT TGG AGG AGC AGT GG 3′ Reverse 5′ GAA GGC ATT TCA TAA GCC TCA 3′.

### 2.10 Immunoblotting

Immunoblotting was performed as previously described (de Sousa Rodrigues et al., 2016) The BCA protein assay (Pierce Scientific, 23225) was used to determine protein concentrations, after which lysates were further diluted 1:1 with 2× Laemmli buffer (BioRad, 1610737) and boiled at 90 °C for 5 min. Samples (10 μg) were electrophoresed using 4–20% Mini-PROTEAN TGX precast gels (BioRad, 4561096) and transferred to 0.45 μm PVDF membranes using the Trans-Blot Turbo System (BioRad, 1704150EDU) according to the manufacturer’s protocol. Membranes were washed and then incubated in 5% powdered milk blocking buffer (BioRad, 1706404) for 1 h before applying primary antibody overnight at 4 °C. The following morning, membranes were briefly washed and incubated at room temperature with HRP-conjugated secondary antibodies for 1 h. Membranes were briefly washed and imaged using Azure Biosystems C400 system or a Li-Cor Odyssey Imaging system to detect chemiluminescent signals. Bands were quantified by densitometry using ImageStudio Lite software. Protein expression was normalized to total protein on a Li-Cor Odyssey instrument (Li-Cor #926-11015).

### 2.11 High-performance liquid chromatography

Levels of dopamine and related analytes in the striatum were measured with electrochemical detection by the Emory HPLC Bioanalytical Core (EHBC) as previously published (Song et al., 2012). Analytes were identified by matching retention time and sensor ratio measures to known standards. Levels of dopamine, DOPAC, HVA, L-DOPA, and 3-methoxytyramine (3-MT) were quantified by comparing peak areas to standards.

### 2.12 RNA sequencing

Total RNA from samples containing the SN was extracted using TRIzol as detailed above. RNA quality, purity, degradation and contamination were assessed by Novogene Corporation (Beijing, China) using a NanoDrop, Agilent 2100, and agarose gel electrophoresis, respectively. cDNA libraries were constructed from RNA samples following the Illumina protocols. Library preparations were sequenced on an Illumina HiSeq 4000 platform at Novogene Corporation. Analysis and KEGG pathway enrichment was completed by Novogene Corporation.

### 2.13 Hematoxylin and eosin staining

For histological analysis, colons were removed, cut longitudinally, Swiss-rolled and flash frozen in optimal cutting temperature (OCT, Tissue-Tek, 4583) embedding medium. Five micromolar sections were cut on the cryostat and placed on charged glass microscopy slides. Hematoxylin and eosin (H&E) staining was performed, and tissue was dehydrated with ethanol and xylene. Images were obtained using a THUNDER microscope (Leica) with a DMC4500 digital camera and LAS X 3D analysis, and 3D visualization advanced software. Images were scored in quadrants. Quadrant scores were calculated based on the sum of inflammatory cell infiltration, epithelial changes, and mucosa architecture, fecal consistency, and fecal blood scores (adapted from Erben et al., 2014). In short, scoring is as follows 1 = minimal inflammatory cell infiltration in the mucosa; minimal epithelial hyperplasia. 2 = Mild inflammatory cell infiltration in the mucosa and submucosa; mild hyperplasia, possible cryptitis and erosion. 3 = Moderate immune cell infiltration; moderate hyperplasia, cryptitis, and possible crypt abscesses with ulcerations in the mucosa. 4 = Marked immune cell inflammation; marked hyperplasia and goblet cell loss; multiple crypts abscesses; extended mucosa ulcerations. The average of each quadrant score represents the final histological score for each sample.

### 2.14 Statistical analysis

Percent body weight changes were analyzed using a two-way analysis of variance (ANOVA) or mixed-effects model with repeated measures on GraphPad Prism 8 software. Data were compared across genotypes using an unpaired student t-test or one-way ANOVA with Tukey’s *post-hoc* for multiple comparisons. Data compared across genotype and treatment were analyzed using a two-way ANOVA with Tukey’s *post-hoc* for multiple comparisons. Significance for all statistical comparisons was set at *p* ≤ 0.05. All data are presented as mean ± SEM. Letters above groups indicate *post-hoc* results. Groups that share the same letter were not significantly different from each other.

## 3 Results

### 3.1 *Lrrk2* G2019S mutant mice exhibited increased inflammatory alterations after acute and chronic colitis

**Figure 1:**
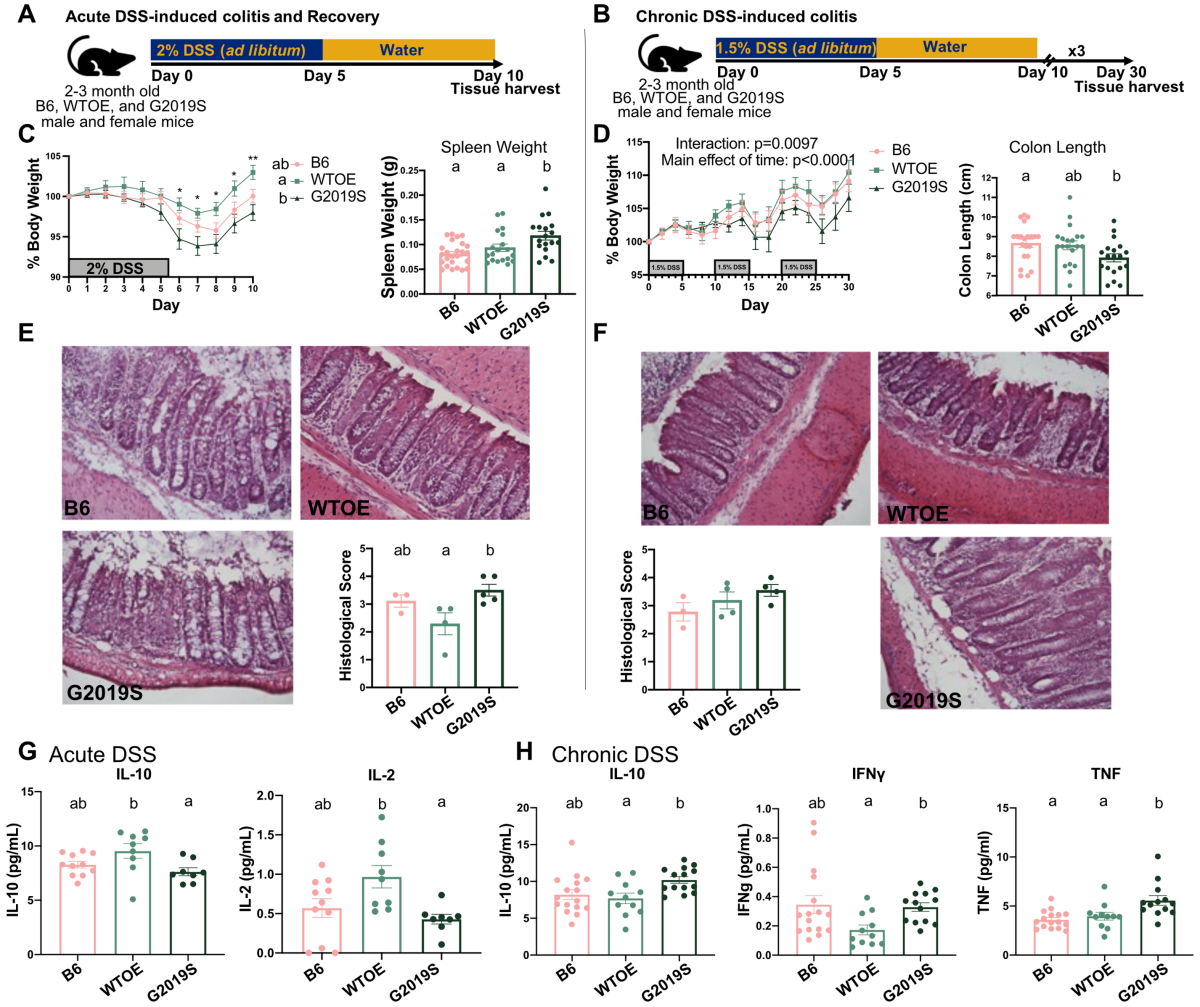
*Lrrk2* G2019S mice were more susceptible than *Lrrk2* WTOE and B6 mice to acute DSS as measured by peripheral cytokine profiles. **(A)** Experimental design for acute DSS-induced colitis and recovery. **(B)** Experimental design for chronic DSS-induced colitis. **(C)** Percent body weight and spleen weight (g) after acute DSS-induced colitis and recovery (*n* = 14–23 per genotype). **(D)** Percent body weight and colon length (cm) after chronic DSS-induced colitis (*n* = 21–26 per genotype). Representative H&E colon images and histological scoring of colon tissue from samples subjected to **(E)** acute DSS-induced colitis and recovery or **(F)** chronic DSS-induced colitis (*n* = 3–5 per genotype). Plasma cytokine levels of **(G)** IL-10 and IL-2 after acute DSS-induced colitis and recovery (*n* = 8–11 per genotype) or **(H)** IFNγ, TNF, and IL-10 after chronic DSS-induced colitis (*n* = 12–17 per genotype) measured by multiplexed immunoassays (MSD). For percent body weight analyses, data were compared across genotypes and time using a mixed-effects model with repeated measures. Asterisks (*) signify differences between WTOE and G2019S groups at that specific time point (*p < 0.05 and **p < 0.01). For all remaining analyses, one-way ANOVA with Tukey’s *post-hoc* for multiple comparisons was used to compare across genotypes. Significance for all statistical comparisons was set at *p* ≤ 0.05. Letters above groups indicate *post-hoc* results. Groups that share the same letter were not significantly different from each other.

To understand the functional significance of increased WT LRRK2 detected in individuals with PD (Cook et al., 2024), we compared BAC G2019S *Lrrk2* mice with wildtype BAC *Lrrk2* overexpressing mice and B6 control mice. Without challenge, 2–3-month-old G2019S and WTOE did not differ from B6 control mice in gross anatomy or peripheral and central immune cell compartments (Supplementary Figure 2). To determine how variability in LRRK2 kinase activity may modulate brain health, we challenged G2019S, WTOE, and B6 mice to both acute and chronic intestinal inflammation. In the acute paradigm, mice were given 2% DSS in their drinking water for 5 days followed by 5 days of water-alone recovery (Figure 1A). For the chronic DSS paradigm, the challenge was repeated three times (Figure 1B). A reduced DSS concentration of 1.5% DSS in the drinking water was used to prevent the mice from reaching humane endpoints. This experimental paradigm models the repeated bouts of inflammation seen in patients with CD. As expected, G2019S mice exhibited increased susceptibility to body weight loss induced by the acute colitis paradigm (Figure 1C); however, no differences in body weight (Figure 1D) were observed in mice on the chronic colitis paradigm. Spleen weight was increased in G2019S mice after acute DSS (Figure 1C), suggesting that mutant G2019S mice displayed increased peripheral immune cell activation. While no differences between genotypes were observed in spleen and colon weight after chronic DSS, mutant G2019S mice exhibited shorter colons (Figure 1D), indicative of greater tissue damage and loss.

DSS exposure denudes epithelial layers in the colon leading to breakdown of the intestinal barrier integrity, mucosal ulceration, and inflammation. Histological analysis of the colon revealed a higher histological score in G2019S mice relative to WTOE mice after acute DSS (Figure 1E) but, surprisingly, no differences after chronic DSS (Figure 1F). Levels of plasma IL-10 and IL-2 were significantly lower in G2019S mice relative to WTOE mice after acute DSS (Figure 1G). In contrast, IL-10, IFNγ, and TNF were increased in the plasma of G2019S mice relative to WTOE mice after chronic DSS (Figure 1H). These seemingly contradictory results may point to impaired early adaptive immune activation which then leads to later compensatory immune escalation. This is in line with previous evidence of the role of LRRK2 in modulating the innate immune response and thus indirectly modifying the chronology of adaptive immune activity.

### 3.2 *Lrrk2* G2019S mice displayed increased signs of neuroinflammation after colitis

**Figure 2:**
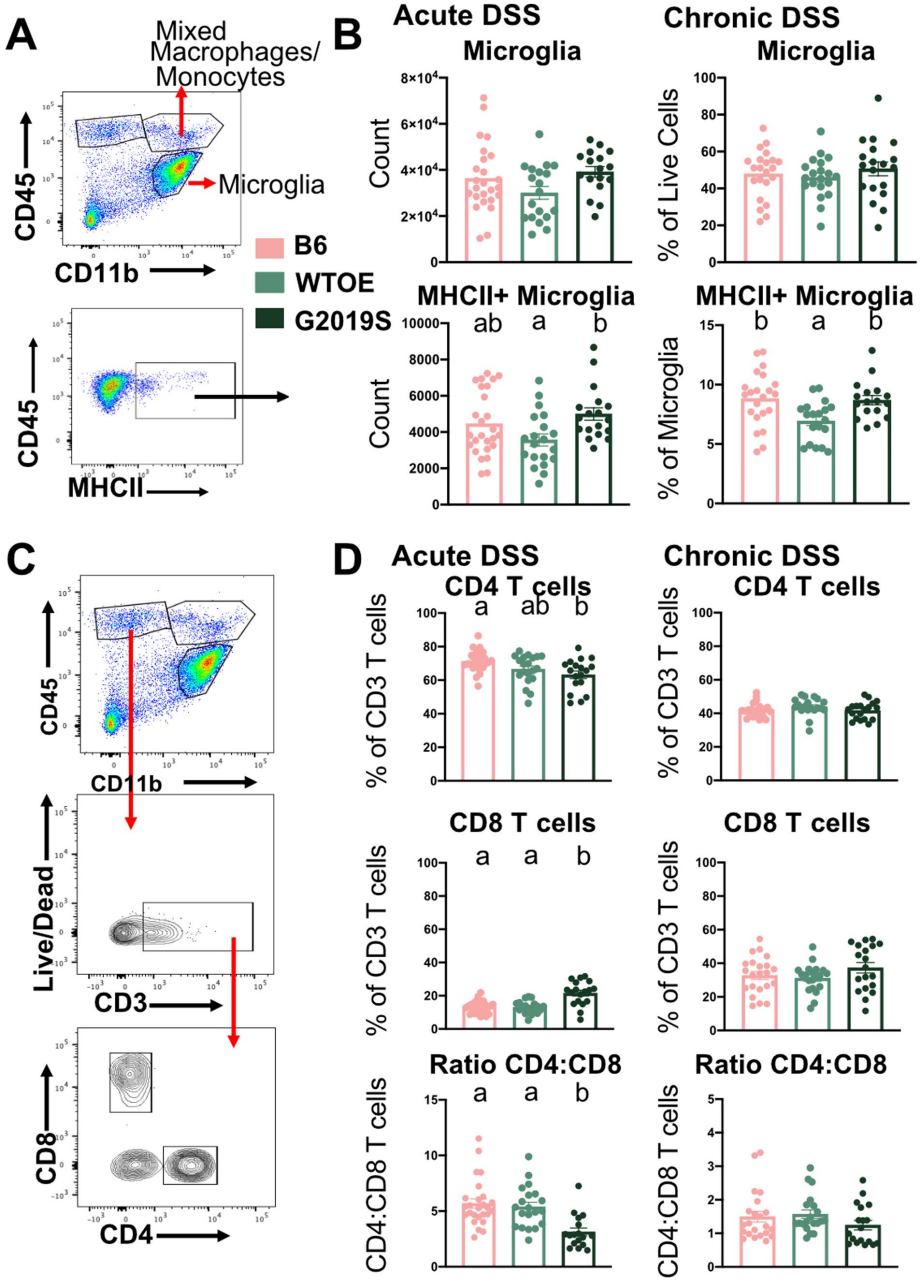
Brain immune cells in BAC *Lrrk2* G2019S mice displayed increased antigen presentation capacity and a shift to cytotoxic T cells compared to WTOE and B6 mice after acute colitis. **(A)** Flow cytometry gating strategy used to identify microglia and MHCII^+^ microglia. **(B)** Quantification of microglia and MHCII^+^ microglia after acute DSS-induced colitis and recovery (*n* = 18–26 per genotype) and chronic DSS-induced colitis (*n* = 19–22 per genotype). **(C)** Flow cytometry gating strategy used to identify T cell populations in the brain. **(D)** Quantification of T cell populations in the brain after acute DSS-induced colitis and recovery (*n* = 18–26 per genotype) and chronic DSS-induced colitis (*n* = 19–22 per genotype). One-way ANOVA with Tukey’s *post-hoc* for multiple comparisons was used to compare across genotypes. Significance for all statistical comparisons was set at *p* ≤ 0.05. Letters above groups indicate *post-hoc* results. Groups that share the same letter were not significantly different from each other.

To assess how increased WT *Lrrk2* or *Lrrk2* G2019S expression might synergize with colitis to impact neuroinflammation, we conducted brain deep-immunophenotyping using multi-color flow cytometry (Figure 2A). We first aimed to understand whether the microglial population changed with treatment. While we observed no differences in the overall number of microglia between genotypes, a significant increase in the number of MHCII^+^ microglia was observed in the brains of BAC G2019S mice relative to WTOE after acute DSS (Figure 2B). In the chronic DSS paradigm we observed no differences in total numbers of cells but found that proportions of MHCII^+^ microglia were smaller in WTOE mice compared to both B6 and G2019S mice (Figure 2B). Like phenotypes observed in PBMCs (Supplementary Figure 3), BAC G2019S mice exhibited a shift in T cell ratios in the brain relative to BAC WTOE mice with higher proportions of CD8 cytotoxic T cells and lower proportions of CD4 T-helper cells after acute DSS (Figure 2C), but these differences were not observed after chronic DSS (Figure 2D). These data indicate that brain immune activation and profiles are shifting during the onset of DSS becomes exacerbated by the G2019S *Lrrk2* mutation in a transient manner.

### 3.3 Chronic DSS altered dopaminergic integrity consistent with nigrostriatal pathway impairment

**Figure 3:**
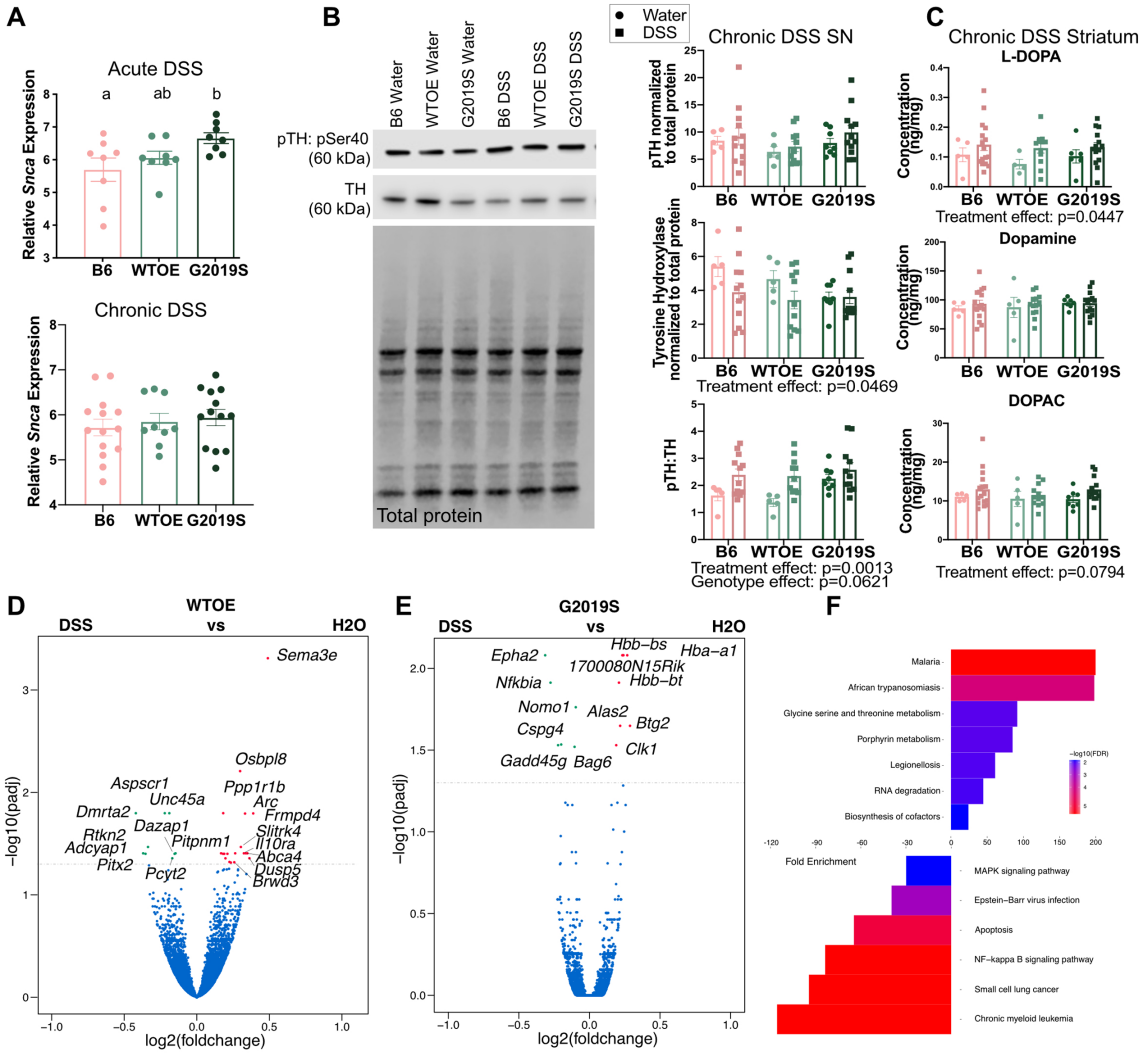
Chronic DSS compromised nigrostriatal pathway integrity regardless of genotype. **(A)** Relative gene expression of *Snca* in the colons of acute or chronic DSS-induced colitis and recovery (*n* = 8–15 per genotype). **(B)** Immunoblot and quantification of nigral pSer40 and TH protein levels after chronic DSS or water (*n* = 5–15 per genotype per treatment). **(C)** Dopamine and its metabolites as measured by HPLC from the striatum of mice treated with chronic DSS or water (*n* = 5–15 per genotype per treatment). Volcano plot showing DEGs in the SN between water and chronic DSS-treated **(D)** BAC WTOE or **(E)** BAC G2019S mice (*n* = 5–15 per genotype per treatment). **(F)** KEGG pathway analysis of genes significantly changing after DSS from G2019S mice. Two-way ANOVA with Tukey’s *post-hoc* for multiple comparisons was used to compare across genotypes and treatments. Significance for all statistical comparisons was set at *p* ≤ 0.05. Letters above groups indicate *post-hoc* results. Groups that share the same letter were not significantly different from each other.

According to the body-first theory of PD pathogenesis, α-synuclein can aggregate in the gut and spread to the brain in a prion-like fashion (Chen and Mor, 2023). We observed genotype-specific increases in *Scna* transcripts by qPCR in the colon of BAC G2019S mice after acute DSS challenge. Surprisingly, no changes in *Scna* transcript were observed after chronic DSS (Figure 3A). To evaluate the effects of colitis on the nigrostriatal pathway, immunoblots of proteins associated with the dopaminergic pathway and measurements of catecholamines by high-performance liquid chromatography (HPLC) were performed. While no differences were found after acute DSS (Supplementary Figure 4), analysis of the nigrostriatal pathway after chronic DSS revealed several significant treatment effects, suggesting that chronic DSS impairs the integrity of dopaminergic neurons independently of genotype. Phosphorylation of tyrosine hydroxylase (TH) at serine 40 is commonly used to assess TH enzymatic activity. Specifically, the ratio of phosphorylated TH to TH (pTH:TH) is used as an indicator of the proportion of active to inactive TH enzyme. Chronic DSS caused a significant reduction in Substantia Nigra (SN) TH protein and an increase in the ratio of phosphorylated TH (pTH) at serine 40 (pSer40) to total TH relative to water controls (Figure 3B). Chronic DSS increased levels of striatal L-3,4-dihydroxyphenylalanine (L-DOPA), the precursor to dopamine, relative to water controls (Figure 3C). No treatment or genotype differences were noted in total dopamine levels, but a trend for DSS to increase 3,4-dihydrozyphenylacetic acid (DOPAC, *p* = 0.0794), one of the metabolites of dopamine, was noted (Figure 3C).

To further parse the effects of chronic DSS on the striatum, we conducted RNA sequencing on BAC WTOE and BAC *Lrrk2* G2019S mice. Twenty-eight differentially expressed genes (DEGs) were identified between WTOE water and chronic DSS-treated mice (Figure 3D; Supplementary material) and 13 DEGs were identified in G2019S mutant mice after DSS-treatment (Figure 3E). The DEGs of the G2019S mice after treatment with DSS translated to 13 enriched KEGG pathways (Figure 3F). Of the KEGG pathways that were differentially enriched, all but one were related to inflammation. MAPK signaling, which has been implicated in frontotemporal dementia (Bottero et al., 2021; Merchak et al., 2026) and Epstein–Barr virus infection, which has been implicated in multiple sclerosis (Bjornevik et al., 2023) were downregulated after DSS. Several KEGG pathways associated with infection were enriched after DSS. Importantly, there was no overlap between the genes that were upregulated after DSS in the WTOE and G2019S mice, indicating that overexpression of mutant *Lrrk2* has measurably different effects on the brain in the context of intestinal inflammation than overexpression of wildtype *Lrrk2*. These data highlight the novel finding that DSS-induced colitis itself has a greater effect on nigrostriatal dysregulation than *Lrrk2* genotype, but that the *Lrrk2* G2019S mutation alters the transcriptional landscape of the inflamed brain.

### 3.4 Acute colitis in aged mice triggered neuroinflammation and compromised nigrostriatal integrity

**Figure 4:**
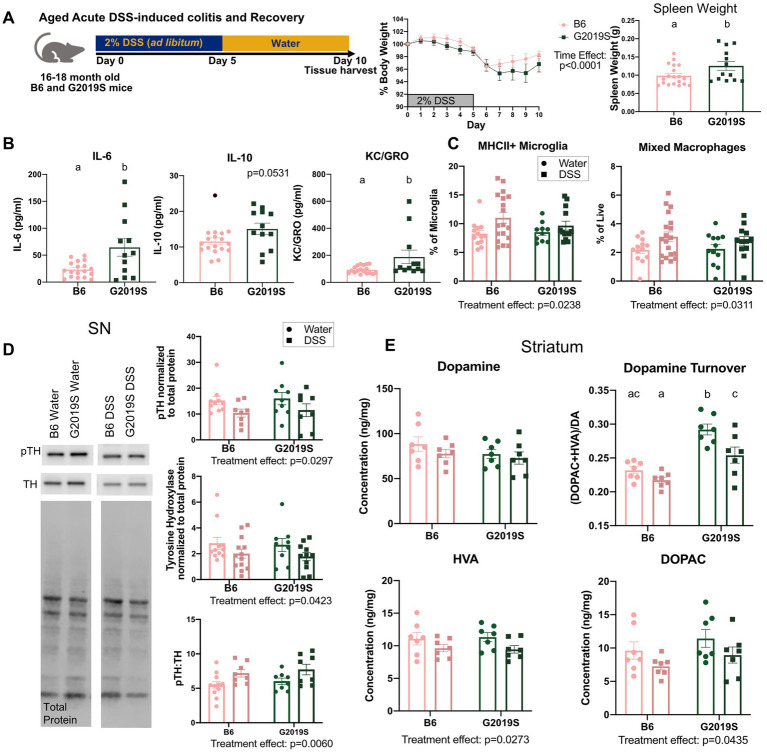
Acute colitis in aged mice was associated with neuroinflammation and compromised nigrostriatal pathway integrity regardless of genotype. **(A)** Experimental design, percent body weight, and spleen weight (g) of aged mice on acute DSS. Data were compared across genotypes and time using a two-way ANOVA with repeated measures or across genotypes with an unpaired student *t*-test (*n* = 12–19 per genotype). **(B)** Plasma cytokine levels of IL-6, IL-10, and KC/GRO of aged mice after acute DSS (*n* = 12–19 per genotype). Data were compared across genotypes with an unpaired student *t*-test. **(C)** Flow cytometry quantification of MHCII^+^ microglia and infiltrating monocytes and macrophages (*n* = 12–19 per genotype per treatment). **(D)** Immunoblot and quantification of nigral pSer40 and TH protein levels (8–11 per genotype per treatment). **(E)** Dopamine, HVA, DOPAC, and dopamine turnover, as measured by HPLC from the striatum of aged mice treated with acute DSS or water (*n* = 7 per genotype per treatment). Two-way ANOVA with Tukey’s *post-hoc* for multiple comparisons was used to compare across genotypes and treatments. Significance for all statistical comparisons was set at *p* ≤ 0.05. Letters above groups indicate *post-hoc* results. Groups that share the same letter were not significantly different from each other.

Given age is the number one risk factor for PD, we sought to assess how *Lrrk2* G2019S-mediated kinase activity might synergize with age and intestinal inflammation. We assessed the same peripheral and CNS parameters as described above in 16–18 month-old B6 and BAC G2019S mice treated with acute DSS. Like the young mice, no differences between G2019S and B6 body weight were observed (Figure 4A). Spleen weight was increased in aged BAC G2019S mice after acute DSS indicating amplified inflammatory profiles (Figure 4A). In accordance, plasma IL-6 and KC/GRO were significantly higher in aged BAC G2019S mice relative to B6 mice (Figure 4B). A non-significant trend for increased plasma IL-10 (*p* = 0.0531) levels was determined in aged BAC G2019S mice relative to B6 (Figure 4B).

Like middle-aged mice, aged mice exhibited a treatment effect with acute DSS increasing the frequency of MHCII^+^ microglia independent of genotype (Figure 4C). However, unlike young mice, aged mice exhibited increased brain-infiltrating macrophages and monocytes after acute DSS independent of genotype as measured by flow cytometry (Figure 4C). No differences between genotype or treatment were noted in T cell populations or total microglia.

In addition to acute DSS inducing neuroinflammation in aged mice, we also observed alterations in the nigrostriatal pathway. Acute DSS caused a significant reduction in TH and pTH protein levels relative to water controls in the SN of aged mice (Figure 4D). This resulted in an increase in the ratio of pTH:TH of DSS-treated mice relative to water controls (Figure 4D). While no differences in dopamine levels were observed, acute DSS decreased the striatal dopamine metabolites, 3,4-dihydroxyphenylacetic acid (DOPAC) and homovanillic acid (HVA), relative to water controls (Figure 4E). Alterations in dopamine turnover were noted with both genotype and treatment effects (Figure 4E). Overall, these data highlight that intestinal inflammation triggers inflammatory and nigrostriatal deficits, but increased LRRK2 protein, whether WT or mutant, has little additional influence on these outcomes regardless of age.

## 4 Discussion

LRRK2 sits at the interface between PD and CD. Increased levels of LRRK2 in PD monocytes (Cook et al., 2017), and inflamed colonic tissue of CD patients (Gardet et al., 2010), suggest a mechanistic link between these two inflammatory conditions. This study directly examined how increased WT or G2019S mutant LRRK2 protein expression influences the gut-brain axis with regard to brain inflammation and nigrostriatal pathway integrity.

We sought to confirm and extend previous studies investigating how LRRK2 levels synergize with intestinal inflammation to promote neuroinflammation and neuropathology associated with PD. While previous studies have explored LRRK2 deficiency (Liu et al., 2011) and WT LRRK2 overexpression in colitis models (Takagawa et al., 2018), to our knowledge, this is the first study to directly compare the neuropathological effects of comparable levels of WT and mutant LRRK2 protein in the context of acute and chronic colitis paradigms. To do so, we utilized BAC transgenic mice overexpressing either wildtype *Lrrk2* or G2019S mutant *Lrrk2*. This model allows us to identify the mutation-specific effects of overexpressed G2019S *Lrrk2* and eliminate the possibility that the observations may simply be due to increased LRRK2 kinase activity. A drawback of this method is that neither mouse model has physiological *Lrrk2* expression levels. For this reason, we also compared each group to standard B6 mice. Consistent with earlier findings showing enhanced susceptibility to acute DSS in BAC WT *Lrrk2* and G2019S knockin mice (Takagawa et al., 2018; Heaton et al., 2025), we observed that BAC G2019S mice also exhibited transient increases in peripheral immune activation following acute colitis. However, we found minimal genotype-specific effects on brain health and inflammation.

Across acute, chronic, and aged paradigms, *Lrrk2* overexpression alone did not alter colon, spleen, or immune cell phenotypes under control conditions. Phenotypic differences emerged only after DSS-induced colitis, reinforcing that peripheral inflammation, not genotype, governs CNS inflammatory and dopaminergic outcomes. Together, these results lead us to reject the hypothesis that *Lrrk2* genotype synergizes with intestinal inflammation to induce brain inflammation or nigrostriatal degeneration. Instead, intestinal inflammation is a much stronger inducer of these brain phenotypes in both middle-aged and older mice independent of genotype. Based on these data, environmental exposure and peripheral inflammation should be viewed as primary targets for prevention of neurodegeneration rather than overfocusing on genetic predisposition.

Although broad genotype effects were limited, subtle and transient immune changes were observed following acute DSS exposure. Specifically, BAC G2019S mice exhibited higher MHCII expression on microglia and a transient shift in CD4:CD8 T-cell ratios favoring CD8^+^ cytotoxic T cells—patterns reminiscent of those seen in PD patient brains (Brochard et al., 2009). These changes were not evident after chronic DSS, consistent with prior work indicating that CD8^+^ T cells act primarily as early initiators of epithelial injury early on in CD but not in remitting bouts of colitis (Cheroutre, 2006; Nancey et al., 2006).

*α*syn is normally expressed in the human enteric nervous system (Böttner et al., 2012; Gray et al., 2014). Pathological forms (aggregated and phosphorylated) of αsyn have been observed throughout the entire GI system of PD patients (Beach et al., 2010) with higher levels of pathological αsyn noted in PD patients relative to healthy controls (Gold et al., 2013; Hilton et al., 2014). An increase in colonic *Snca* mRNA following acute DSS in BAC G2019S mice further suggests transient activation of enteric immune signaling, consistent with prior reports showing elevated α-synuclein transcripts after intestinal inflammation (Choi et al., 2018). Importantly, these changes were absent after chronic DSS and were not accompanied by detectable differences in nigral or striatal α-synuclein pathology, underscoring that mouse α-synuclein lacks the aggregation-prone properties characteristic of the human protein (Volles and Lansbury, 2007; Cookson, 2009). Future studies should explore the effects of WT versus mutant *Lrrk2* in colitis models in humanized αsyn mice.

Assessment of the nigrostriatal pathway revealed no genotype-specific effects under any DSS condition, but significant treatment-dependent alterations following chronic DSS. Increased pTH:TH ratios and elevated L-DOPA levels in DSS-treated mice indicate compensatory activation of dopamine synthesis, an early hallmark of dopaminergic stress preceding overt neuron loss. Although dopamine concentrations were unchanged, these findings are consistent with early-stage dysfunction rather than established degeneration (Barrio et al., 1990; Sossi et al., 2002). In conclusion, these findings collectively demonstrate that intestinal inflammation and age—rather than *Lrrk2* mutation status—are the predominant drivers of neuroinflammatory and dopaminergic dysfunction in this model. While *Lrrk2* genotype modestly modulates specific immune parameters, it does not exacerbate colitis-induced neuroinflammation or nigrostriatal compromise. These results reinforce the need to focus on environmental and inflammatory triggers as central targets for prevention and treatment of nigrostriatal degeneration.

## Data Availability

The datasets presented in this study can be found in online repositories. The names of the repository/repositories and accession number(s) can be found at: https://doi.org/10.5281/zenodo.18351472.
