# Supplementary material for: Intestinal inflammation promotes neuroinflammation and PD-associated nigrostriatal pathology independently of LRRK2 G2019S kinase activity
Source: Front Cell Neurosci. 2026 Apr 10;20:1794784. doi: 10.3389/fncel.2026.1794784 (PMC13105876; doi:10.3389/fncel.2026.1794784)
Supplement: Supplementary file 3 [file Supplementary_file_1.docx]

Supplementary Material

# Supplementary Figures

**Supplementary Figure 1.** Flow cytometry gating strategies.

**Supplementary Figure2.** Analysis of genotype effects on BAC WTOE, BAC G2019S, and B6 mice at baseline. Mice serve as controls throughout the manuscript **(A)** Percent body weight change after 10 days of no treatment (n=18-29 per genotype). **(B)** Spleen weight, colon length, and colon weight of the mice from A. Gating strategy and flow cytometry read outs of the **(C)** PBMCs and **(D)** brain of untreated mice. For percent body weight analyses, data were compared across genotypes and time using a mixed-effects model with repeated measures. Asterisks (*) signify differences between WTOE and G2019S groups at that specific time point (*p<0.05, **p<0.01). For all remaining analyses, one-way ANOVA with Tukey’s *post hoc* for multiple comparisons was used to compare across genotypes. Significance for all statistical comparisons was set at p≤0.05. Letters above groups indicate *post hoc* results. Groups that share the same letter were not significantly different from each other.

**Supplementary Figure 3.** Flow cytometric analysis of T cells in the peripheral blood of young mice exposed to either acute or chronic DSS. **(A)** Gating strategy. Flow cytometric results from **(B)** acute DSS and **(C)** chronic DSS. One-way ANOVA with Tukey’s *post hoc* for multiple comparisons was used to compare across genotypes. Significance for all statistical comparisons was set at p≤0.05. Letters above groups indicate *post hoc* results. Groups that share the same letter were not significantly different from each other.

**Supplementary Figure 4.** Western blots for TH and α-syn in the striatum of young mice after acute DSS. One-way ANOVA with Tukey’s *post hoc* for multiple comparisons was used to compare across genotypes. Significance for all statistical comparisons was set at p≤0.05. Letters above groups indicate *post hoc* results. Groups that share the same letter were not significantly different from each other.
